# A Scalp Tumor Revealing Metastatic Breast Cancer: A Case Report

**DOI:** 10.7759/cureus.93299

**Published:** 2025-09-26

**Authors:** El Hammouti Mohamed, El Achchi Anass, Kharkhach Ayoub, Tariq Bouhout, Badr Serji

**Affiliations:** 1 Department of Surgical Oncology, Regional Oncology Center, Mohammed VI University Hospital, Oujda, MAR; 2 Department of Surgical Oncology, Faculty of Medicine and Pharmacy, Mohammed First University, Oujda, MAR

**Keywords:** breast cancer, carcinoma, cutaneous, metastases, scalp

## Abstract

Scalp metastases in invasive ductal carcinoma of the breast are extremely rare, occurring in less than 1% of patients. In large retrospective studies of cutaneous metastases, only a small proportion of patients with solid tumors develop skin involvement, with breast cancer being the most common source.

We report a case of a patient who developed a single metastatic lesion involving the subcutaneous scalp tissue and the median frontal bone, with intracranial extension reaching the right temporo-occipital region, detected 11 years after the initial diagnosis and treatment of the primary breast tumor. This case highlights the importance of considering atypical metastatic sites during long-term follow-up of breast cancer survivors. It discusses the diagnostic and therapeutic challenges posed by such unusual presentations.

## Introduction

Invasive ductal carcinoma of the breast is the most common malignant tumor in women. Its prognosis largely depends on several factors, including tumor stage, histological grade, and immunohistochemical markers, such as hormone receptor status and HER2 expression, as well as the presence or absence of metastases. Scalp metastases in breast invasive ductal carcinoma are extremely rare, typically seen in less than 1% of patients [1]. In large retrospective studies of cutaneous metastasis, only about 0.3% of solid tumor patients manifest skin involvement, with breast cancer being the most common source [2].

The most frequent metastases of breast cancer often develop in the lungs, bones, liver, and brain. Bone metastases are particularly common, affecting approximately 70% of patients with metastatic breast cancer. The most frequently involved bones are the ribs, spine, pelvis, and long bones in the arms and legs [3]. The most commonly affected sites for cutaneous metastases of breast carcinoma include the chest wall and abdomen, accounting for approximately 60-70% of cases. Still, they can also occur in the head and neck region and extremities [4]. Distant isolated cutaneous metastases are uncommon in breast carcinoma. Furthermore, a single isolated metastasis in the scalp is extremely rare in this type of cancer, as observed in the present case.

Cutaneous metastases from breast cancer may present as nodules or plaques that are flesh-colored, pink-red, or violaceous, or as areas of patchy hair loss, either isolated or multiple, which can be mistaken for various dermatological conditions. We report a case of a patient who developed a single metastatic lesion involving the subcutaneous scalp tissue and the median frontal bone, with intracranial extension, occurring 11 years after the diagnosis and treatment of the primary breast tumor.

## Case presentation

We present a case of a 55-year-old female patient with no significant medical history and no family history of breast cancer. She presented with a palpable breast lump in the upper outer quadrant (UOQ) of the left breast, incidentally discovered 11 years ago during self-examination. She reported no associated symptoms, such as pain, nipple discharge, or systemic manifestations.

Her menstrual and obstetric history was unremarkable. There were no known risk factors for breast cancer. Physical examination revealed a firm, mobile nodule measuring approximately 1 cm in the left UOQ, with no overlying skin changes; palpable axillary lymph nodes were noted. Initial radiological evaluation, including mammography and breast ultrasound, confirmed a suspicious mass. There was no evidence of distant metastases at the time of diagnosis. The tumor was staged as T1N1M0 (stage II) according to the American Joint Committee on Cancer (AJCC) Eighth Edition.

The patient underwent a total mastectomy with axillary lymph node dissection. Histopathological examination revealed a 1 cm residual tumor located 3 cm from the deep resection margin and 6 cm from the lateral resection margin. Nineteen lymph nodes were dissected, with eight positive nodes (8/19). The tumor was histologic grade II, and immunohistochemistry showed ER+, PR+, HER2-. The surgical procedure was followed by adjuvant chemotherapy consisting of three cycles of doxorubicin (60 mg/m²) combined with cyclophosphamide (600 mg/m², days 1-21), followed by three cycles of paclitaxel (175 mg/m² every 21 days). This was complemented with adjuvant radiotherapy, delivering 50 Gy to the left supraclavicular region and 50 Gy to the left chest wall. Subsequently, the patient was placed on hormone therapy with tamoxifen (20 mg/day) for five years, achieving complete clinical remission, and remained disease-free until lost to follow-up.

**Figure 1:**
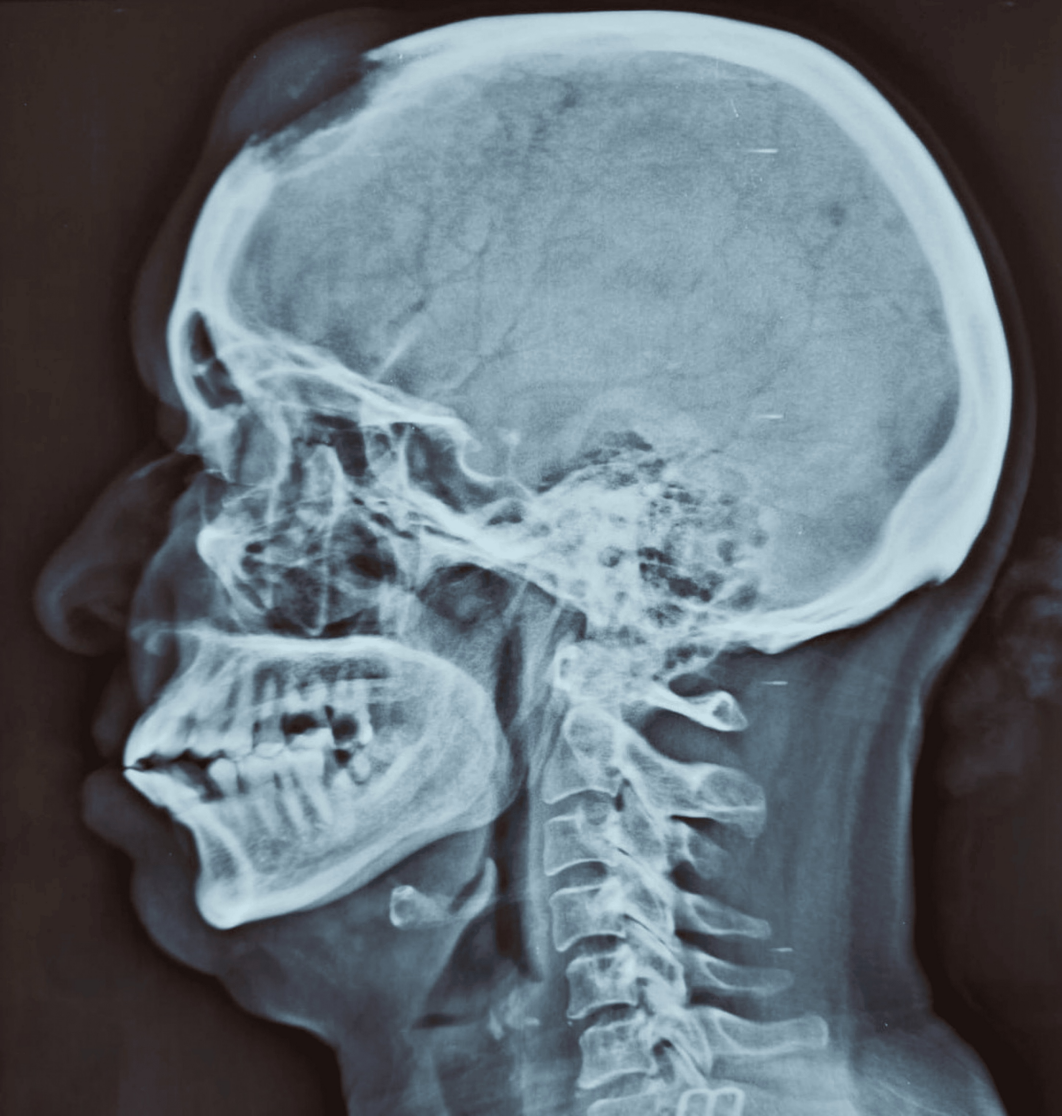
Lateral skull radiograph demonstrating a lytic lesion involving the median frontal bone with extension toward the subcutaneous scalp tissue.

Eleven years after the initial diagnosis and treatment of the primary breast tumor, corresponding to six years after discontinuing hormonal therapy, the patient presented with a progressively enlarging frontal scalp mass of 4 cm in diameter, which was noticed approximately three months prior to consultation. The mass was associated with localized pain and intermittent headaches, but no neurological deficits, seizures, or systemic symptoms. On physical examination, the lesion was located in the median frontal scalp region, firm in consistency, non-pulsatile, and slightly tender to palpation. The overlying skin was intact, without ulceration, discharge, or local inflammatory changes. The mass appeared fixed to the underlying bone, with no regional lymphadenopathy (Figure 1).

**Figure 2:**
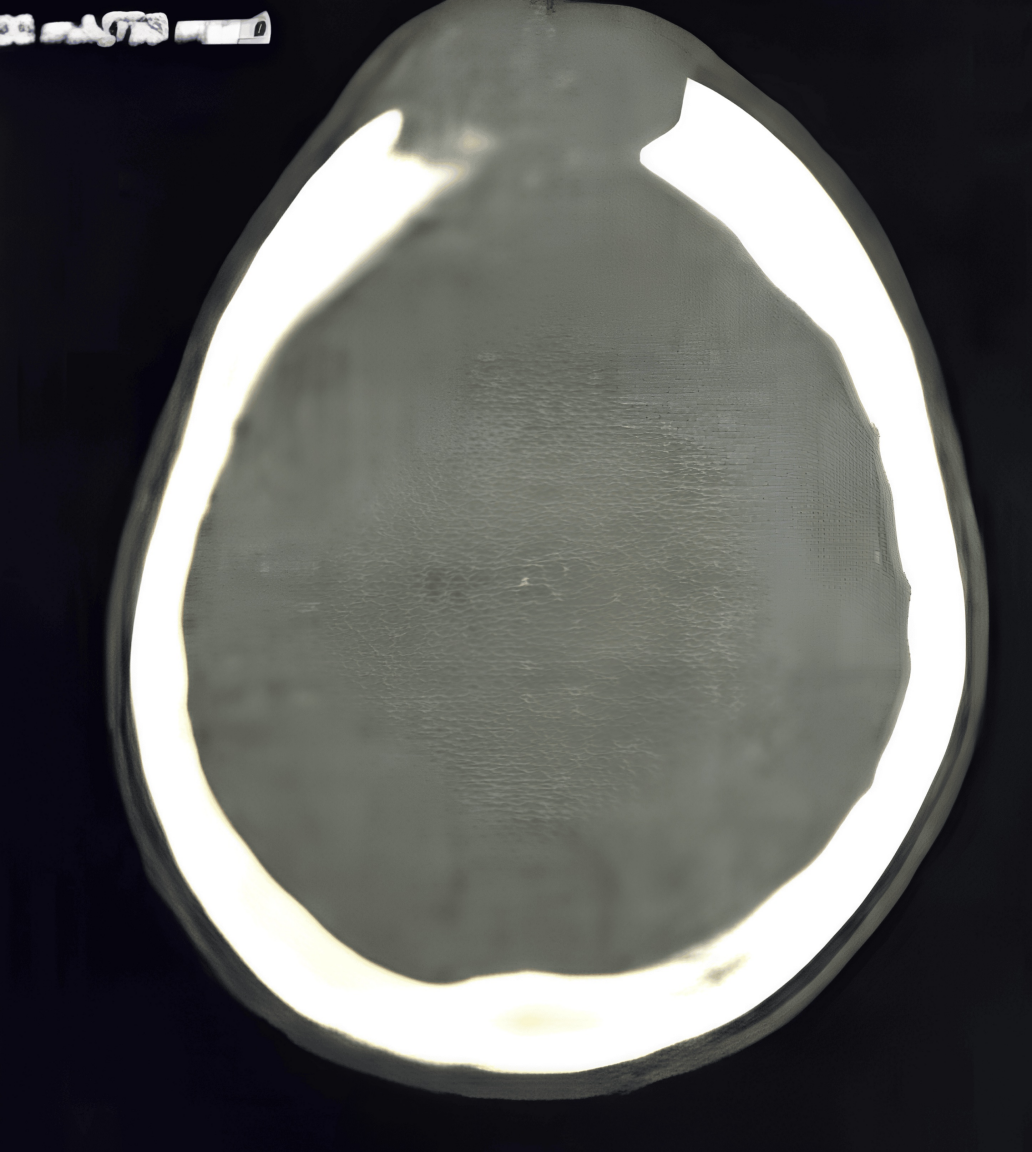
Axial non-contrast brain CT scan showing an osteolytic lesion of the medial frontal osteolytic tissue lesion with the beginning of endocranial extension.

**Figure 3:**
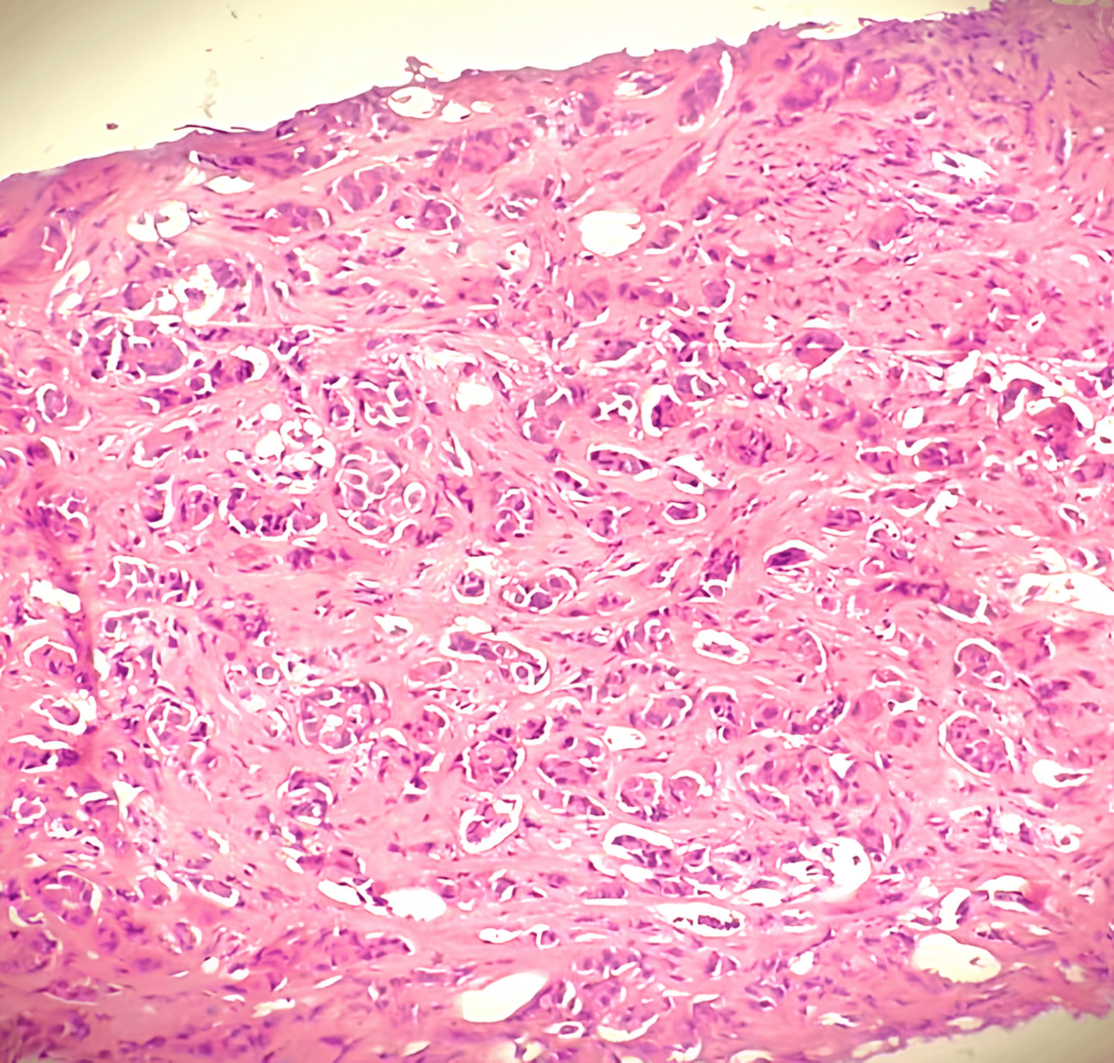
Photomicrograph showing secondary infiltration by an infiltrating mammary type adenocarcinoma.

A brain CT scan revealed a median frontal osteolytic tissue lesion, measuring approximately 4 cm in greatest diameter, with early intracranial extension, associated with a right temporo-occipital osteolytic lesion measuring approximately 3×2×2 cm, suggesting a secondary origin (Figure 2). The patient underwent a Tru-Cut biopsy of the mass, which revealed secondary infiltration by an invasive breast adenocarcinoma. According to the AJCC Eighth Edition, the disease was staged as stage IV metastatic breast carcinoma (Figure 3).

Following tumor board discussion, the patient underwent urgent palliative radiotherapy delivering a total dose of 30 Gy in 10 fractions of 3 Gy each. Given her immunohistochemistry (IHC) profile (ER+, PR+, HER2-, Ki-67 30%), immunotherapy was not indicated. She was subsequently started on first-line palliative chemotherapy with paclitaxel (80 mg/m²) administered on days one, eight, and 15 for three cycles.

The clinical course was marked by a partial regression of the soft tissue component of the cranial lesion, with an estimated 75% reduction in size on follow-up CT. Additional radiotherapy was not indicated due to prior dosing and concurrent systemic progression. The patient later developed further osseous metastases and a malignant pleural effusion, consistent with the natural course of advanced metastatic breast carcinoma. The follow-up CT imaging demonstrated an increased number of osteolytic bone metastases, with a denser appearance of pre-existing lesions, indicating progression of metastatic disease, as well as the development of a moderate left-sided pleural effusion.

Consequently, the patient was initiated on second-line chemotherapy with capecitabine (Xeloda, 1000 mg × 2/m²) and received zoledronic acid (4 mg every three months). Despite these interventions, her disease progressed with recurrent pleural effusion and additional skeletal lesions. She was subsequently managed with supportive palliative care but succumbed to her illness approximately 10 months after the diagnosis of metastatic recurrence.

## Discussion

Breast carcinoma is one of the most frequently metastasizing solid tumors. The most common metastases of breast cancer often occur in the lungs, bones, liver, and brain [5]. Cutaneous metastases from breast cancer are relatively uncommon, occurring in approximately 2.5-10% of patients. Despite their relative rarity, breast cancer is the most frequent solid tumor to metastasize to the skin, accounting for about 30% of all cutaneous metastases from solid tumors [6].

Cutaneous metastases usually develop after the initial diagnosis of the primary malignancy; however, in some cases, they may represent the first indication of an otherwise undiagnosed visceral tumor. The development of cutaneous metastases in breast cancer is a complex process influenced by multiple factors. Several mechanisms have been proposed, including direct extension from underlying structures, hematogenous spread via the bloodstream, and lymphatic dissemination. Molecular characteristics of the tumor, such as hormone receptor status, HER2 expression, and other genetic alterations, may influence the tropism of cancer cells for skin tissue. Additionally, interactions between tumor cells and the microenvironment of the skin, including adhesion molecules, chemokine receptors, and the extracellular matrix, facilitate colonization and growth at cutaneous sites [7].

The most frequently affected areas for cutaneous metastases are the chest wall and, less commonly, the scalp. This distribution is related to the lymphatic drainage pathways of the breast, which primarily flow toward the axillary, internal mammary, and supraclavicular lymph nodes. Tumor cells can disseminate through these lymphatic channels to the overlying skin. Scalp involvement, although rare, may occur through retrograde spread via the cervical and occipital lymphatic networks, as well as through hematogenous routes facilitated by the scalp's rich vascularization [8].

According to a study examining metastatic patterns in breast cancer, cutaneous metastases accounted for 7.1% of cases in invasive ductal carcinomas and 6.5% in invasive lobular carcinomas among all metastatic sites [9]. Breast cancer accounts for approximately 33% of all cutaneous metastases, highlighting its increased tendency to involve the skin compared to other malignancies. Other solid tumors that commonly give rise to cutaneous metastases include lung carcinoma, colorectal carcinoma, melanoma, ovarian carcinoma, and renal cell carcinoma. However, the overall frequency is lower than in breast cancer [8]. Scalp tumors are rare, representing approximately 2% of all cutaneous tumors. They are often diagnosed at advanced stages of cancers, such as lung and stomach cancers, and more rarely, breast cancer [10].

The detection of cutaneous metastases typically reflects advanced disease and is associated with a poor prognosis, as hematogenous dissemination of circulating tumor cells drives metastatic spread. Management of these patients is primarily palliative, aiming to control symptoms and improve quality of life. Therapeutic options include systemic therapies, such as hormone therapy, chemotherapy, targeted therapy, or immunotherapy, depending on tumor subtype and molecular profile. Local treatments, including palliative radiotherapy or surgical excision, may be considered for symptomatic or isolated lesions. Supportive care addressing pain, ulceration, and infection is also essential [8].

A study conducted by Chiu et al. on scalp lesions revealed that 12.8% of these lesions were metastatic [11]. Lung cancer was the most frequently observed primary tumor, followed by liver, colon, and breast cancer. Breast cancer origin was the least common, accounting for approximately 7.84% of cases [12]. The main differential diagnosis for breast cancer metastases to the scalp is primary adenocarcinoma of the cutaneous appendages (sweat glands), which must be distinguished through histological examination and immunohistochemistry [13]. Histopathological analysis is critical for establishing the diagnosis. Systemic staging can be assessed using imaging techniques, particularly computed tomography (CT).

Therapeutic options for cutaneous metastases include surgery, radiotherapy, systemic therapy, and palliative care, tailored to the progression of the disease and the patient’s overall condition. In our patient, due to the advanced stage of disease with scalp and calvarial metastasis and intracranial extension, surgical excision was not feasible. She underwent urgent palliative radiotherapy to the cranial lesion, followed by first-line chemotherapy with paclitaxel, and later second-line chemotherapy with capecitabine, complemented by zoledronic acid for bone metastases. Supportive care was also provided to manage pain and symptomatic complications [8].

## Conclusions

Distant cutaneous metastases of breast cancer, particularly on the scalp, are very rare. They may represent the only visible sign of disease progression or metastatic dissemination. The presence of a scalp nodule in a patient with a history of breast cancer should raise suspicion for metastasis at this site. However, only a biopsy with immunohistochemical analysis can differentiate it from a cutaneous tumor. After histological confirmation, management can be adjusted, such as through the administration of local chemotherapy, radiotherapy, or surgical resection, depending on the patient’s overall condition.
